# Genomic Analysis of Multidrug-Resistant *Mycobacterium tuberculosis* Strains From Patients in Kazakhstan

**DOI:** 10.3389/fgene.2021.683515

**Published:** 2021-11-09

**Authors:** Asset Daniyarov, Askhat Molkenov, Saule Rakhimova, Ainur Akhmetova, Dauren Yerezhepov, Lyailya Chingissova, Venera Bismilda, Bekzat Toksanbayeva, Anar Rakisheva, Ainur Akilzhanova, Ulan Kozhamkulov, Ulykbek Kairov

**Affiliations:** ^1^ Laboratory of Bioinformatics and Systems Biology, Center for Life Sciences, National Laboratory Astana, Nazarbayev University, Nur-Sultan, Kazakhstan; ^2^ Laboratory of Genomic and Personalized Medicine, Center for Life Sciences, National Laboratory Astana, Nazarbayev University, Nur-Sultan, Kazakhstan; ^3^ National Scientific Center of Phthisiopulmonology of the Republic of Kazakhstan, Almaty, Kazakhstan; ^4^ Department of Phthisiopulmonology, School of General Medicine, Asfendiyarov Kazakh National Medical University, Almaty, Kazakhstan

**Keywords:** tuberculosis, genomic analysis, whole genome, genetic variants, *Mycobacterium tuberculosis*, multidrug resistant TB, MIRU-VNTR analysis, spoligotyping

## Abstract

Tuberculosis (TB) is an infectious disease that remains an essential public health problem in many countries. Despite decreasing numbers of new cases worldwide, the incidence of antibiotic-resistant forms (multidrug resistant and extensively drug-resistant) of TB is increasing. Next-generation sequencing technologies provide a high-throughput approach to identify known and novel potential genetic variants that are associated with drug resistance in *Mycobacterium tuberculosis* (*Mtb*). There are limited reports and data related to whole-genome characteristics of drug-resistant *Mtb* strains circulating in Kazakhstan. Here, we report whole-genome sequencing and analysis results of eight multidrug-resistant strains collected from TB patients in Kazakhstan. Genotyping and validation of all strains by MIRU-VNTR and spoligotyping methodologies revealed that these strains belong to the Beijing family. The spectrum of specific and potentially novel genomic variants (single-nucleotide polymorphisms, insertions, and deletions) related to drug resistance was identified and annotated. ResFinder, CARD, and CASTB antibiotic resistance databases were used for the characterization of genetic variants in genes associated with drug resistance. Our results provide reference data and genomic profiles of multidrug-resistant isolates for further comparative studies and investigations of genetic patterns in drug-resistant *Mtb* strains.

## Introduction

Tuberculosis (TB) is an infectious disease that remains a significant global health problem. TB is responsible for 1.4 million deaths annually (World Health Organization, 2020). Approximately one-third of the world’s population has been infected with multidrug-resistant *tuberculosis* (MDR-TB) and MDR-TB is a growing problem globally. Among the 30 high MDR-TB burden countries reported by the World Health Organization (WHO), Kazakhstan (population 19 million; www.worldometers.info/world-population/kazakhstan-population/) ranks eighth in the world, with approximately 12,300 (2016) total new and relapse TB cases (World Health Organization, 2017).

Kazakhstan is one of the countries with a high burden of MDR-TB resistant to rifampicin (MDR/RR-TB), with a total incidence rate of 22 cases per 100,000. During the last 10 years, the TB incidence and mortality rate in Kazakhstan has decreased, but the MDR-TB incidence rate is still at a high stable level (World Health Organization, 2020). MDR-TB is caused by *Mycobacterium tuberculosis (Mtb)* resistant to at least the two basic first-line anti-TB drugs (i.e., rifampicin and isoniazid). In Kazakhstan, MDR/RR-TB comprises 27% of new TB cases and 44% of previously treated TB cases. In 2019, there were an estimated 4,100 incident cases of MDR/RR-TB (World Health Organization, 2020).

Treatment of patients with MDR-TB and extensively drug-resistant TB (XDR-TB) requires the use of more toxic and expensive chemotherapy drugs and prolonged hospitalization. Treatment is often ineffective, which consequently results in high disability and mortality. Thus, the high incidence and mortality rate of MDR-TB complicates efforts to combat this disease.

Whole-genome investigations using next-generation sequencing (NGS) are implemented in many fields of biomedical research, particularly infectious diseases. NGS technologies allow analysis of whole-genome sequences of infectious pathogens and investigations of their molecular and genetic features. NGS also facilitates the development of the fundamental and practical foundations of microbiology, virology, and epidemiology (Barzon et al., 2011; Koser et al., 2012). Identification of features and differences in *Mtb* genomes provides an understanding of the nature of drug resistance and the evolutionary processes of clinical isolates and assists in the study of TB outbreaks and virulence factors of individual strains.

There are currently limited reports on whole-genome sequencing of MDR *Mtb* human clinical isolates from Kazakhstan. It is useful and extremely important to examine drug-resistant *Mtb* strains with different mutations in genes encoding drug metabolism among clinical isolates. Thus, the present study was designed to evaluate and to characterize mutations associated with drug resistance to first- and second-line anti-TB drugs among MDR *Mtb* isolates from Kazakhstan and to present phylogenetic analysis results of *Mtb* based on genome data.

## Materials and Methods

### Clinical Isolates and Drug Susceptibility Test

Eight multidrug-resistant isolates of *Mtb* from TB patients in Kazakhstan with pulmonary and extrapulmonary TB were collected and analyzed. Multidrug-resistant *tuberculosis* (MDR-TB) is defined as “resistant to at least isoniazid and rifampin, the two most effective first-line TB drugs.” After primary isolation, *Mycobacteria* were subcultured on solid Löwenstein–Jensen (LJ) medium as described previously (World Health Organization, 1998). Drug-susceptibility testing (DST) of *Mtb* was performed by the absolute concentration method on solid LJ medium containing 40 μg/ml of rifampicin or 0.2–1 μg/ml of isoniazid according to WHO recommendations (World Health Organization, 2003) and by using the BACTEC-MGIT 960 Mycobacteria Growth Indicator Tube (BD Diagnostic Systems, United States) system according to the instructions of the manufacturer for isoniazid, rifampicin, streptomycin, and ethambutol. Results of microbiological studies on solid LJ medium were recorded 28 days after culturing. Isolates were considered resistant if more than 20 colonies appeared on media containing anti-TB drugs. Identification and isolation of pure culture of the pathogen was performed at the Reference Laboratory of National Scientific Center of Phthisiopulmonology of the Republic of Kazakhstan in Almaty.

### Genotyping of *M. tuberculosis* Isolates

The collected isolates were subjected to DNA spoligotyping by a standard method (Kamerbeek et al., 1997) using a reverse dot-blot spoligotyping commercially available kit (Ocimum Biosolutions Inc.) with positive (*M. tuberculosis* H37Ra, and *M. bovis*) and negative controls. Hybridization of PCR fragments on the membrane with chemiluminescent detection and further analysis was performed. The presence or absence of a signal in binary format was recorded for each clinical isolate of *Mtb*, where for each of the 43 spacer intervals of direct repeats (DR) region, one (1) indicates the presence of a hybridization signal and zero (0) for its absence. The obtained spoligotyping data were compared using published SITVIT web (Institute Pasteur de Guadeloupe) database (http://www.pasteur-guadeloupe.fr:8081/SITVIT_ONLINE/query) (Demay et al., 2012). Spoligotypes not assigned to any clades with the SITVIT WEB database were compared with the MIRU-VNTRplus database (Allix-Béguec et al., 2008). All eight isolates had the Beijing spoligotype (000000000003771) based on spoligotyping analysis results.

MIRU-VNTR (mycobacterial interspersed repetitive unit-variable number of tandem repeats) typing using 12 loci was performed as previously described (Supply et al., 2006). Known primers were used for 24 MIRU-VNTR loci from the database (http://www.miru-vntrplus.org/MIRU/).

Genome data of eight MDR *Mtb* isolates were used for IS6110-based restriction fragment length polymorphism (RFLP) typing analysis. Analysis of the resolving ability of CDC-standard (Centers for Disease Control and Prevention) *Mtb* fingerprinting (spoligotyping plus 24 loci MIRU-VNTR typing) was conducted by using the Comprehensive Analysis Server for the *Mycobacterium tuberculosis* complex (CASTB) (Iwai et al., 2015). All isolates with «Beijing » spoligotype were placed in «Lineage 2 (East-Asian lineage)».

### DNA Extraction and Whole-Genome Sequencing

Material collection and DNA extraction from sputum were performed for eight *Mtb* isolates assigned as *Mtb*-MDR-KZ (1280), *Mtb*-MDR-KZ (1405), *Mtb*-MDR-KZ (1410), *Mtb*-MDR-KZ (1524), *Mtb*-MDR-KZ (1525), *Mtb*-MDR-KZ (1577), *Mtb*-MDR-KZ (1585), and *Mtb*-MDR-KZ (1713). DNA was extracted from *Mtb* cultures by the cetyl-trimethyl ammonium bromide (CTAB) method (Van Soolingen et al., 1994). DNA quality and quantity were assessed by a Qubit 3.0 Fluorometer with dsDNA Broad Range Assay kit (Thermo Fisher Scientific) and agarose gel electrophoresis. DNA libraries were prepared using a GS FLX Titanium rapid library preparation kit. Prepared genomic libraries of the eight MDR-TB strains were sequenced on a Roche 454 GS FLX + Titanium NGS platform at the Center for Life Sciences, Nazarbayev University, Kazakhstan.

### Genome Analysis and Variant Calling

Raw sequencing data generated from Roche 454 GS FLX + Titanium NGS platform in sff format were converted to fastq format using SFF converter (Galaxy tool v.1.0.1, https://usegalaxy.org/). FastQC (Andrews, 2014) was applied to analyze read quality and adapters were trimmed with Trimmomatic v0.38 software (Bolger et al., 2014) to truncate low-quality reads, filtering for a minimum read length of 36 (parameter: LEADING: 3; TRAILING: 3; SLIDINGWINDOW: 4:20; MINLEN: 36; CROP: 120) and trim low-quality 3′ ends of reads. Nucleotide positions in the reads with a quality score lower than Q20 were removed. The resulting sequence data were deposited at NCBI Sequence Read Archive under accession number PRJNA503963. Mapping statistics were generated by QUAST v.5.0 (Gurevich et al., 2013) (Table 1). Gene prediction and annotation were performed using Genome Annotation Service PATRIC (Wattam et al., 2014).

**TABLE 1:** Whole-genome mapping and sequencing statistics of eight Kazakhstan *Mycobacterium tuberculosis* isolates.

Isolate name	Total raw paired read	N50	Completion (%)^a^	Full length (bp)	Coverage (×)^a^	SNPs^*^	InDels^*^	GC (%)	Genes^b^	tRNAs	rRNAs
*Mtb*-MDR-KZ (1280)	195,048,344	90,951	97.14	4 331 459	51×	1,621	247	65.53	4,385	44	3
*Mtb*-MDR-KZ (1405)	126,653,863	50,264	96.99	4 331 257	34×	1,600	246	65.54	4,388	44	3
*Mtb*-MDR-KZ (1410)	89,936,746	23,343	96.38	4 332 751	24×	1,374	200	65.52	4,457	44	3
*Mtb*-MDR-KZ (1524)	160,749,277	51,640	96.94	4 332 192	43×	1,597	234	65.55	4,388	44	3
*Mtb*-MDR-KZ (1525)	118,335,745	37,986	96.80	4 328 788	31×	1,594	239	65.55	4,395	44	4
*Mtb*-MDR-KZ (1577)	120,396,884	44,245	96.91	4 327 338	33×	1,601	244	65.53	4,399	44	3
*Mtb*-MDR-KZ (1585)	85,923,603	18,929	96.20	4 334 662	23×	1,312	204	65.52	4,446	44	3
*Mtb*-MDR-KZ (1713)	113,788,645	35,572	96.76	4 328 294	31×	1,582	243	65.55	4,385	44	3

Note. ^a^Computed against the respective *M. tuberculosis* reference strain H37Rv (NC_000,962; 4,411,532 bp) and after-read quality control.

b Total genes (coding genes, RNA, genes, and pseudogenes) annotated using PATRIC.

*Genetic variants identified by Samtools.

To identify genetic differences between MDR-TB isolates, the reference *Mtb* H37Rv genome was used for variant calling. For each isolate, the sequence reads were aligned against the *Mtb* H37Rv reference genome using BWA 0.6.2 (Li and Durbin, 2009a). Samtools (Li et al., 2009b) toolkit was applied for identification of single-nucleotide polymorphisms (SNPs) and insertions and deletions (InDels). The Samtools/mpileup pipeline is provided in Supplementary File S1. Annotation of SNPs and InDels was conducted by the GMTV database (Chernyaeva et al., 2014). Identified genomic variants were annotated using an in-house prepared R script (Supplementary File S2).

### Identification of Gene Mutations Associated With Drug Resistance

All isolates were subjected to DST of five first-line drugs (isoniazid, INH; rifampicin, RIF; streptomycin, SM; ethambutol, EMB; and pyrazinamide, PZA) using three databases—ResFinder (Zankari et al., 2012), CARD (Mcarthur et al., 2013), CASTB (Iwai et al., 2015), TGS-TB tool (Sekizuka et al., 2015), and TB-Profiler (Phelan et al., 2019) as an initial screening method. Sequences were analyzed for the presence of antimicrobial resistance (AMR) genes and genomic assemblies using ResFinder v.3.2 (with database updated on October 1, 2019) (Kaas et al., 2014) including PointFinder v.3.1.0 (Mi et al., 2018) (with 98% threshold for identity with the reference and minimum 60% coverage of the gene length; database updated on July 2, 2019) and CASTB web server with default settings. Raw FASTQ sequencing files were uploaded to TGS-TB, an online tool to determine drug resistance *in silico* (Sekizuka et al., 2015). TGS-TB was used to analyze the mutations involved in drug resistance and related to lineage determination.

### Phylogenetic Analysis

Nine *Mtb* reference strain genomes were used for comparative genomics analysis, namely, H37Ra (NZ_CP016972) (Levaditi et al., 1950), H37Rv (NC_018143) (Cole et al., 1998), K (NZ_CP007803) (Han et al., 2015), CDC1551 (NC_002755) (Valway et al., 1998), KZN 4207 (NC_016768) (Ioerger et al., 2009), PanR1006 (NZ_CM002097) (Lanzas et al., 2013), XDR KZN 605 (Uddin et al., 2018), strain RUS B0/W148 (NZ_CP030093) (Mokrousov et al., 2015), and str. Beijing/NITR203 (NC_021054) (Narayanan and Deshpande, 2013) (Supplementary File S3). Comparative phylogenetic analyses was performed by the CSI Phylogeny tool (options: minimum depth, 10x; minimum relative depth, 10%; minimum distance between SNPs, 10 bp; minimum SNP quality, 30; minimum read mapping quality, 25; minimum Z-score, 1.96) (Kaas et al., 2014) from whole-genome sequences based on the high-quality SNPs.

### Enrichment Analysis

Gene enrichment analyses with Fisher exact and binomial test options were performed on common non-synonymous SNPs and InDels using the PANTHER Classification System (Mi et al., 2018), which provides a comprehensive set of efficient and concise annotation tools for researchers to understand the biological meaning behind numerous genes. A false discovery rate (FDR) < 0.05 was set as the cutoff criterion. All data analysis and visualization of variants were conducted using R 3.5.1.

## Results

This study was a whole-genome sequencing investigation to present in-depth whole-genome data and genetic variations in loci associated with drug resistance in selected Kazakhstani clinical strains of MDR-TB. The results of phylogenetic analysis of *Mtb* based on genome data are presented.

All eight multidrug-resistant clinical isolates of *Mtb* from TB patients with pulmonary and extra-pulmonary TB were collected at the National Research Center for Phthisiopulmonology (Kazakhstan). Of the eight MDR-TB patients, seven were male and one was female; six patients were identified as smear-positive and two patients were smear-negative. Age distribution of TB patients revealed that patients were young and of reproductive age; the youngest was 14 years, and oldest patient was 38 years. The median age of those with MDR-TB was 26 years. The most frequently diagnosed TB form was infiltrative pulmonary TB (five patients), followed by cavernous pulmonary TB (two patients) and extrapulmonary TB (one patient, skeletal bones TB).

Whole-genome sequencing for the eight MDR-TB clinical isolates produced 667,052,381 paired reads with an average read length of 520 bp. For all libraries sequenced, over 98% of the reference genome (H37Rv, NC_018,143) was covered by at least one read and an average depth of coverage of 31 × was achieved. Whole-genome mapping and sequencing statistics are shown in Table 1. The average sequencing coverage of isolates ranged from 21 × to 51 × and mapping quality ranged from 96.2 to 97.14%. The average number of predicted genes (4,405), GC content (65.54%). The number of SNPs and InDels ranged from 1,312 to 1,621 and from 200 to 247, respectively.

### Mutations Associated With Drug Resistance

Among all isolates, we detected several new genetic variants in drug-resistance genes that were not described or were unknown in antibiotic resistance databases (ResFinder v.3.2; CARD; CASTB) (Table 2). Genetic variants in multiple genes associated with drug resistance in *Mtb* were identified from whole-genome sequencing data (Supplementary File S4). SNPs in each of the *Mtb* genomes were identified against the *Mtb* H37Rv reference using an in-house developed analysis pipeline. A total of eight isolates had mutations in genes associated with resistance to INH and FLQ, including *katG* S315T and *katG* R463L, respectively. The eight mutations (*rpoB*: p. S450L, *gyrA*: p. E21Q, *katG:* p. S315T, *gidB*: p. E92D, *embB*: p.M306V, *katG*: p. R463L, *gyrA*: p. G668D, *gyrA*: p. S95T) were found in all MDR-TB isolates. RIF resistance-conferring mutations were identified in the rpoB gene for all eight MDR isolates (100%). Only *Mtb*-MDR-KZ (1,410) isolate had an RIF resistance double mutation at the rpoC gene (codon 698) and rpoB gene. The *Mtb*-MDR-KZ (1,713) isolate with mutations at the *fabG1* promoter site was predicted to have co-resistance to INH, EMB, RIF and SM (Supplementary File S4).

**TABLE 2:** Drug-resistance profiling of eight multidrug-resistant *tuberculosis* (MDR-TB) isolates.

Isolate	Drug resistance analysis									Microbiology
Sample ID	AMK	EMB	INH	OFX	PZA	RIF	SM	KM	CM	
*Mtb*-MDR-KZ (1280)	S/S/S/S	R/R/R/R	R/R/R/R	S/S/S/S	S/S/R/S	R/R/R/R	S/R/S/R	S/S/S/S	S/S/S/S	INH–R; RIF–R EMB–R; SM–S
Mtb-MDR-KZ (1405)	S/S/S/S	R/R/R/R	R/R/R/S	S/S/S/S	R/S/S/S	R/R/R/S	R/R/R/R	S/S/S/S	S/S/S/S	INH–R; RIF–R EMB–R; SM–R
*Mtb*-MDR-KZ (1410)	S/S/S/S	R/R/R/R	R/R/R/R	S/S/S/S	S/S/S/S	R/R/R/R	R/R/R/S	S/S/S/S	S/S/S/S	INH–R; RIF–R EMB–R; SM–R
*Mtb*-MDR-KZ (1524)	S/S/S/S	R/R/R/R	R/R/R/R	S/S/S/S	R/S/S/S	R/R/R/S	R/R/R/R	S/S/S/S	S/S/S/S	INH–R; RIF–R EMB–R; SM–R
*Mtb*-MDR-KZ (1525)	S/S/S/S	R/R/R/R	S/R/R/S	S/S/S/S	R/S/S/S	R/R/R/S	R/R/R/S	S/S/S/S	S/S/S/S	INH–R; RIF–R EMB–R; SM–R
*Mtb*-MDR-KZ (1577)	S/S/S/S	R/R/R/R	R/R/R/R	S/S/S/S	R/S/R/S	R/R/R/S	R/R/R/R	S/S/S/S	S/S/S/S	INH–; RIF–R EMB–R; SM–R
*Mtb*-MDR-KZ (1585)	R/R/R/S	R/R/R/S	R/R/R/S	S/S/S/S	S/-/S/S	R/R/R/S	R/R/R/S	R/R/S/S	R/R/S/S	INH–R; RIF–R EMB–R; SM–R
*Mtb*-MDR-KZ (1713)	S/S/S/S	R/R/R/R	R/R/R/S	S/S/S/S	R/S/S/R	R/R/R/S	R/R/R/S	S/S/S/S	S/S/S/S	INH–R; RIF–R EMB–R; SM–R

Note. *1/2/3/4, ResFinder 3.2/CARD/CASTB/TGS-TB.

First-Line: I, isoniazid (INH); R, rifampicin (RIF); S, streptomycin (SM); E, ethambutol (EMB); P, pyrazinamide (PZA).

Second-Line: A, amikacin (AMK); O, ofloxacin (OFX); K, kanamycin (KM); C, capreomycin (CM).

By closely examining the correlation of the phenotypic drug susceptibility profiles of the strains with mutations identified in their drug resistance-associated genes, we identified potential new genetic determinants of drug resistance. For example, only [isolate *Mtb*-MDR-KZ (1,405)] had the *gyrB* D461N mutation, which is known to confer resistance to fluoroquinolones (FLQ). All eight isolates had the same *gyrA* E21Q, G668D, and S95T mutations seen in FLQ-susceptible strains, indicating that these mutations are not the source of FLQ resistance. For EMB, all isolates (100%) showed mutations in the *embB* gene, with the mutation Met306Val being the most common. PZA resistance-conferring mutations were identified in five isolates (62.5%). All mutations occurred as a single mutation in the *pncA* gene. For FLQ, mutations in *katG* codon 463 were identified in eight isolates (100%). Regarding resistance to SM and injectable agents, mutations were found in the *rpsL* loci in eight isolates (100%). Mutation at rrs codon 1,401 was only found in a poly-resistant strain.

### Single-Nucleotide Polymorphism Clustering and Distribution in *Mycobacterium tuberculosis* Genomes

Further comparative genomic analysis identified a total of 1,933 non-repetitive SNPs, among which a common pool of 1,037 SNPs was shared by the eight isolates (Supplementary File S5).

The total number of identified SNPs (point mutations differing from H37Rv) ranged from 1,312 to 1,621 (mean 1,535). Of the SNPs in coding regions, 65% were considered nonsynonymous substitutions yielding a mean nonsynonymous/synonymous ratio (Ns/S) of 1.63. Overall, across the eight clinical isolates and nine publicly available reference strains (H37Ra, H37Rv, K, CDC1551, KZN 4207, PanR1006, XDR KZN 605, strain RUS B0/W148, str. BeijingNITR203), genomic SNPs were identified by mapping to the reference genome of *Mtb* H37Rv. The number of small InDels detected upon read mapping ranged from 200 to 247 InDels per isolate with a size between 1 and 136 bp (Table 3).

**TABLE 3:** Characteristics of genetic variants of MDR-TB isolates: data derived from WGS including mapping indicators.

Isolate	SNPs									InDels^a^		Mapping indicator^b^
	Nonsynonymous mutations (Ns)				Synonymous mutations (S)	Total in coding regions (Tc)	Total	N_s_/S ratio	T_c_/total	Total	Size range	Coverage (%)
	Total	In PPE genes	In PE genes	In all other								
*Mtb*-MDR-KZ (1280)	890	93	129	668	550	1,360	1,621	1.6181	0.8390	247	136	97.14
*Mtb*-MDR-KZ (1405)	895	95	126	674	547	1,364	1,600	1.6362	0.8525	246	136	96.99
*Mtb*-MDR-KZ (1410)	751	71	99	706	469	1,176	1,374	1.6013	0.8559	200	69	96.38
*Mtb*-MDR-KZ (1524)	890	96	122	672	546	1,355	1,597	1.6300	0.8485	234	136	96.94
*Mtb*-MDR-KZ (1525)	879	95	109	675	549	1,350	1,594	1.6011	0.8469	239	136	96.80
*Mtb*-MDR-KZ (1577)	887	111	113	663	532	1,339	1,601	1.6673	0.8363	244	136	96.91
*Mtb*-MDR-KZ (1585)	732	88	63	581	453	1,139	1,312	1.6159	0.8681	204	61	96.20
*Mtb*-MDR-KZ (1713)	884	97	112	675	528	1,343	1,582	1.6742	0.8490	243	117	96.76

Note. ^a^Small InDels identified using Samtools.

b Relative to *M. tuberculosis* H37Rv.

PE, genes, genes with an N-terminal proline-glutamine motif; PPE, genes, genes with an N-terminal proline-glutamine-glutamine motif.

### Phylogenetic Analysis of *Mycobacterium tuberculosis* Isolates

An NJ phylogenetic tree was created based on SNPs from whole-genome sequences of the eight clinical *Mtb* isolates and the other nine reference *Mtb* strains. Completely sequenced genomes of nine *Mtb* strains available through NCBI were selected for comparative analysis (Supplementary File S3). The phylogenetic tree was built based on the SNPs by the CSI Phylogeny tool. GS Reference Mapper was used to obtain the map-based genome sequence in FASTA format and further phylogenetic analysis.

The analysis showed that the first cluster (*n* = 7) is closely related to RUS B0/W148 strain sequenced in the Russian Federation, and the last clinical isolate from another obtained cluster (*n* = 1) *Mtb*-MDR-KZ (1405) is phylogenetically comparable with str. BeijingNITR203, which was isolated in India.

**FIGURE 1:**
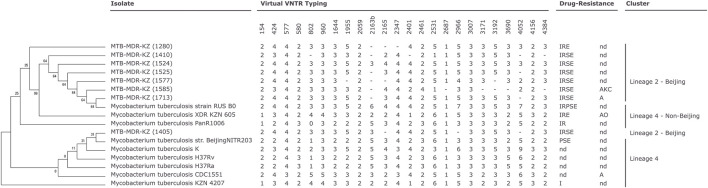
Phylogenetic relationships of *Mycobacterium* (*M*)*. tuberculosis* isolates based on high-quality single-nucleotide polymorphisms (SNPs) identified by CSI Phylogeny tool (Kaas et al., 2014) from whole-genome sequences. The tree was constructed by the neighbor-joining method. Mycobacterial interspersed repetitive unit-variable number of tandem repeats (MIRU-VNTR) genotyping analysis of the 17 *M. tuberculosis* isolates (Supplementary Data S3). MIRU-VNTR dendrogram of the eight multidrug-resistant (MDR) *M. tuberculosis* clinical isolates. First-line drug susceptibility testing: I, isoniazid; R, rifampicin; S, streptomycin; E, ethambutol; P, pyrazynamid. Second-line drug susceptibility testing: K, kanamycin; A, amikacin; C, capreomycin; O, ofloxacin; nd, not determined; -, not determined number of tandem repeats (virtual VNTR typing).

Phylogenetic analysis and *in silico* VNTR revealed that the isolates were distributed into “modern” lineage (lineage 2) (Figure 1). The East Asian (L2–Beijing) sub-lineage was predominant and was found in all cases.

### Spoligotyping Results of Mycobacterial Interspersed Repetitive Unit-Variable Number of Tandem Repeats

All eight clinical isolates of *Mtb* in this study were phenotypically resistant to RIF and INH and can be characterized as MDR-TB. DNA was used in genotyping by the 12-loci MIRU-VNTR method. VNTR typing of the eight MDR-TB isolates recovered from patients with *tuberculosis* in Kazakhstan using 12 polymorphic loci identified two clusters. *Mtb* H37Rv reference strain was used as a positive control, and sterile ultrapure water was used as a negative control for MIRU-VNTR genotyping. All MDR-TB strains belonged to the W-Beijing family with the genetic MIRU-VNTR digital profiles 223325153533 (*n* = 6) and 223325173533 (*n* = 2). Six strains had a profile (223325153533) corresponding to a major Asian/Russian subtype, Beijing/M2 clusters 94–32, which is prevalent in Kazakhstan. Two of eight strains belonging to Beijing/M11 clusters 100–32 (223325173533) are prevalent in China and Russia.

According to spoligotyping results, all eight clinical isolates of *Mtb* belonged to the W-Beijing family (East Asian lineage) and have the same spoligo-octal code (000000000003771, SIT#1).

Genotyping technique grouped the eight MDR-TB isolates and nine *Mtb* reference strains into two major clades: Beijing Lineage 2 and Beijing Lineage 4 (Figure 1, Supplementary File S6).

### Enrichment Analysis

Gene Ontology (GO) analysis is a useful method for annotating genes and gene sets with biological characteristics for high-throughput genome data. We identified 302 nonsynonymous SNPs and InDels common among all nine MDR-TB isolates that were used in further downstream GO analysis. We analyzed differences in the enrichment of GO categories using the PANTHER Classification System (Mi et al., 2018). The results of the PANTHER analysis are presented in Supplementary File S7. Functional annotations of proteins encoded by genes showed significantly (*p* < 0.05) increased or decreased enrichment (Supplementary File S8) with classification according to their associated biological processes and molecular functions. GO function annotation for 288 different genes was performed and sorted based on *p*-values, and enriched pathways were determined (Supplementary File S8). The selected genes were categorized according to GO classification based on their hypothetical molecular functions and biological processes. GO enrichment analyses demonstrate that organic substance metabolic process and cellular process are primarily enriched in biological processes, while molecular function pathway analysis indicates that ion binding and catalytic activity are mainly enriched (Supplementary File S7).

## Discussion

In this study, we report whole-genome sequencing characterization of MDR-TB clinical isolates circulating in Kazakhstan. We sequenced and analyzed eight *Mtb* isolates and provide genetic insights into genes associated with drug resistance in comparison with phenotypic tests.

Several studies on biological diversity and identification of mutations in specific genomic regions encoding drug resistance were performed in Kazakhstan (Kozhamkulov et al., 2011; Ibrayeva et al., 2014; Akhmetova et al., 2015; Skiba et al., 2015). Only a few publications related to whole-genome sequencing of clinical isolates performed by next-generation sequencing platforms have briefly reported draft genome sequences of *Mtb* strains circulating in Kazakhstan (Ilin et al., 2015; Kairov et al., 2015; Daniyarov et al., 2020).

In a 2015 study, Skiba et al. (2015) described the population structure and phylogeography of *Mtb* in Kazakhstan and the influence of determined genotypes on the distribution of drug-resistant strains. A total of 151 *Mtb* isolates from different regions of the country were genotyped by MIRU-VNTR and spoligotyping. Results of genotyping revealed that the Beijing genotype was the most widely distributed family (*n* = 109) in Kazakhstan. In 2005, an association of the Beijing genotype with drug resistance in Kazakhstan was shown first time by Kubica et al. (2005). W-Beijing family strains were also a prevalent genotype and were found in 78.4% of cases among pyrazinamide-susceptible and pyrazinamide-resistant *Mtb* clinical isolates (Akhmetova et al., 2015) and 68.3% of clinical isolates from TB patients in the penitentiary system belonged to the Beijing family (Ibrayeva et al., 2014). Thus, W-Beijing strains were the most widespread family in previous studies in Kazakhstan.

Strains of the Beijing family were identified for the first time during MDR-TB outbreaks in the 1990s in New York (USA). Later, in 1995, *Mtb* isolates with the same characteristics were found in Beijing (China), and this group of isolates was named “Beijing” (Glynn et al., 2002; Mitchell, 2002). The Beijing genotype is currently distributed across almost all continents. Strains of the Beijing family are more virulent, and most are associated with drug resistance. Using high-resolution typing methods (RFLP and MIRU-VNTR), clinically or epidemiologically significant clusters within drug-resistant Beijing family strains were identified, such as strain W that caused the MDR-TB outbreak in New York in the 1990s, the highly transmissible Gran Canarian strain GC1237 with ongoing transmission, and two large clusters circulating in the former Soviet Union republics (named 94–32 and 100–32) (Yin et al., 2016).

In a study from the European Centre for Disease Prevention and Control on the 2014 Asian/Russian cluster, Beijing 94–32 was identified in 23.7% of MDR-TB cases in 14 European countries, while the Russian type Beijing 100–32 was found in fewer countries (11 European countries) with more cases of MDR-TB (33.6%) (European Centre for Disease Prevention and Control, 2016). These two clusters were identified among the reported eight MDR-TB clinical isolates reported here. The first Asian/Russian cluster, Beijing/M2 94–32 is the largest and dominant subtype in Kazakhstan and is prevalent throughout the former USSR. The second cluster, Beijing/M11 100–32, is prevalent in Russia, China, and the East Asia region.

Phylogenetic analysis based on genome data of the eight MDR (*Mtb*-KZ) *Mtb* strains in comparison with nine susceptible/drug-resistant strains (H37Ra, H37Rv, K, CDC1551, KZN 4207, PanR1006, XDR KZN 605, strain RUS B0/W148, str. BeijingNITR203) revealed clustering to two main clades. Phylogenetic analysis showed one cluster, including seven strains and one Beijing family strains. Phylogenetic analysis showed that one cluster that included seven strains was closely related to strain RUS B0/W148 (W-Beijing), and the last clinical isolate was closely related to Beijing NITR203, which were initially identified in neighboring countries Russia and India, respectively.

Analysis of mutations in genes involved in RIF and INH resistance of *Mtb* strains isolated in Kazakhstan revealed that the most frequent mutations are substitutions at codons 531 and 315 of the *rpoB* and *katG* genes, respectively (Hillemann et al., 2005; Kozhamkulov et al., 2011; Ibrayeva et al., 2014). In 2005, analysis of the *rpoB* gene for the presence of mutations in the hot-spot region among 92 MDR isolates showed mutations at five codons (531, 526, 516, 522, and 514) responsible for RIF resistance; all MDR clinical isolates carried a mutation at codon 315 of the *katG* (Hillemann et al., 2005). Among 272 RIF-resistant clinical isolates from a study in 2011, mutations were detected in 255 isolates (93.7%) as 13 different variants in four codons (Ser531, Asp516, His526, and Leu533). INH-resistant *Mtb* isolates (*n* = 310) were studied, and resistance-causing mutations in *katG* and the promoter regions of fabG-inhA and oxyR-ahpC were identified (Kozhamkulov et al., 2011).

According to previous studies, mutations at codon 531 in *rpoB* and codon 315 in *katG* are predominant among multidrug-resistant strains and clinical isolates that mostly belong to the Beijing family in Kazakhstan (Hillemann et al., 2005; Kozhamkulov et al., 2011; Ibrayeva et al., 2014).

Thus, this study provides insight into the resistance of MDR *Mtb* strains circulating in Kazakhstan using whole-genome sequencing. All eight clinical isolates of *Mtb* in this study were phenotypically resistant to RIF and INH and were, thus, characterized as MDR-TB. The obtained genomic data of *Mtb* strains were compared with whole-genome sequencing data and annotations using the CARD, CASTB, ResFinder v.3.2, and TB-Profiler databases. The obtained variants were analyzed following the known genes that confer resistance to each anti-TB drug. The results of whole-genome sequencing revealed known and unknown mutations in the genes that cause resistance to first- and second-line anti-TB drugs, where amino acid substitutions were noted in the target genes.

MIRU-VNTR analysis and spoligotyping of clinical isolates of *Mtb* revealed a pattern associated with W-Beijing (East Asian lineage), which is prevalent in Kazakhstan and other countries in Central Asia. All of the MDR-TB strains were the part of the W-Beijing family divided by MIRU-VNTR typing into two clusters, namely, Asian/Russian, Beijing/M2 94–32, which is the largest and dominant subtype in Kazakhstan (*n* = 6), and Beijing/M11 100–32 (*n* = 2), which is prevalent in the East Asian region and Russia.

Phylogenetic analysis and *in silico* VNTR revealed that all eight MDR *Mtb* isolates were distributed into “modern” lineage (lineage 2). This W-Beijing sub-lineage East Asian (L2–Beijing) was found in all eight cases.

In the current study, resistance mutations to all first-line anti-TB drugs, FLQs, and second-line injectable drugs were observed. Second-line drug resistance was observed in one isolate. We revealed the changes in genetic regions associated with drug resistance to first- and second-line anti-TB drugs using the databases ResFinder 3.2 and TB profiler (Supplementary File S4). Known mutations with amino acid substitutions described in the ResFinder 3.2/TB profiler databases and unknown/novel mutations in genomes of the MDR-TB were identified. The number of genes where unknown/novel mutations were detected varied from 6–10 genes among the eight MDR-TB clinical isolates, and the number of amino-acid substitutions ranged from 9–70. The number of genes where unknown/novel mutations were determined from both databases ranged from 1–4 genes, and the number of amino acid substitutions ranged from 1–6. The results obtained require further additional studies and can be used as additional sources for a more detailed study of new target regions of the *Mtb* genome associated with drug resistance.

Thus, high-throughput genome sequencing and comprehensive analysis from eight MDR-TB clinical isolates revealed important information about the nature of drug resistance and phylogenetic relationships of isolated strains in Kazakhstan. This study reported the use of next-generation sequencing technology for whole-genome annotation of clinical MDR-TB isolates from Kazakhstan, showing its potential for clinical management and TB control. According to the results of whole-genome sequencing, known and putative mutations in genes that cause resistance to first- and second-line anti-TB drugs were found, and amino acid substitutions in target genes were identified.

Our study has several limitations. We were not able to collect *M. tuberculosis* clinical isolates from different regions of Kazakhstan and perform genome sequencing for the larger number of clinical isolates. For this initial study, we included only eight well-characterized MDR clinical isolates provided by the National Reference Lab for genomic analysis. This study is a pilot study with limited samples and will require an increased sample size for further large-scale WGS studies. It will be useful to expand this early investigation to include not only MDR samples but also other DR samples from different regions of Kazakhstan in future study.

These predicted findings and identified putative genetic variants can be validated by further experimental studies and functional investigations. A growing array of rapid, reliable, and increasingly accessible technologies of whole-genome sequencing is aimed at all components of TB control, such as diagnosis, treatment, and investigation of infection sources. Whole-genome sequencing of individual *Mtb* strains and predicting their drug-resistance profiles, particularly against first-line anti-TB drugs, allows rapid and appropriate treatment initiation and control of drug resistance. Generated and analyzed whole-genome data may serve as reference background for comparative studies among different isolates and future TB clinical management and surveillance in Kazakhstan, Central Asian region, and neighboring states.

## Data Availability

The resulting sequence data were deposited at National Center for Biotechnology Information Sequence Read Archive under accession number PRJNA503963.

## Abbreviations

BACTEC-MGIT, mycobacteria growth indicator tube; CDC, Centers for Disease Control and Prevention; CASTB, Comprehensive Analysis Server for the Mycobacterium tuberculosis complex; CTAB, cetyl-trimethyl ammonium bromide; DST, drug susceptibility testing; DR, direct repeats; GO, Gene Ontology; InDels, insertions and deletions; LJ, Löwenstein–Jensen; Mtb, Mycobacterium tuberculosis; MIRU-VNTR, mycobacterial interspersed repetitive unit-variable number of tandem repeats; MDR-TB, multidrug-resistant tuberculosis; MDR/RR-TB, multidrug- and rifampicin-resistant tuberculosis; NGS, next-generation sequencing; RFLP, restriction fragment length polymorphism; SNPs, single-nucleotide polymorphism; TB, tuberculosis; WHO, World Health Organization; XDR-TB, extensively drug-resistant tuberculosis.

## References

[B1] Akhmetova A. Kozhamkulov U. Bismilda V. Chingissova L. Abildaev T. Dymova M. (2015). Mutations in the pncA and rpsA Genes Among 77 *Mycobacterium tuberculosis* Isolates in Kazakhstan. Int. J. Tuberc. Lung Dis. 19, 179–184. 10.5588/ijtld.14.0305 25574916

[B2] Allix-Béguec C. Harmsen D. Weniger T. Supply P. Niemann S. (2008). Evaluation and Strategy for Use of MIRU-VNTR Plus , a Multifunctional Database for Online Analysis of Genotyping Data and Phylogenetic Identification of *Mycobacterium tuberculosis* Complex Isolates. J. Clin. Microbiol. 46, 2692–2699. 10.1128/JCM.00540-08 18550737 PMC2519508

[B3] Andrews S. (2014). FastQC A Quality Control Tool for High Throughput Sequence Data.

[B4] Barzon L. Lavezzo E. Militello V. Toppo S. Palù G. (2011). Applications of Next-Generation Sequencing Technologies to Diagnostic Virology. Ijms 12, 7861–7884. 10.3390/ijms12117861 22174638 PMC3233444

[B5] Bolger A. M. Lohse M. Usadel B. (2014). Trimmomatic: a Flexible Trimmer for Illumina Sequence Data. Bioinformatics 30, 2114–2120. 10.1093/bioinformatics/btu170 24695404 PMC4103590

[B6] Chernyaeva E. N. Shulgina M. V. Rotkevich M. S. Dobrynin P. V. Simonov S. A. Shitikov E. A. (2014). Genome-wide *Mycobacterium tuberculosis* Variation (GMTV) Database: a New Tool for Integrating Sequence Variations and Epidemiology. BMC Genomics 15, 308. 10.1186/1471-2164-15-308 24767249 PMC4234438

[B7] Cole S. T. Brosch R. Parkhill J. Garnier T. Churcher C. Harris D. (1998). Deciphering the Biology of *Mycobacterium tuberculosis* from the Complete Genome Sequence. Nature 393, 537–544. 10.1038/31159 9634230

[B8] Daniyarov A. Molkenov A. Rakhimova S. Akhmetova A. Nurkina Z. Yerezhepov D. (2020). Whole Genome Sequence Data of *Mycobacterium tuberculosis* XDR Strain, Isolated from Patient in Kazakhstan. Data in Brief 33, 106416. 10.1016/j.dib.2020.106416 33102665 PMC7578676

[B9] Demay C. Liens B. Burguière T. Hill V. Couvin D. Millet J. (2012). SITVITWEB - A Publicly Available International Multimarker Database for Studying *Mycobacterium tuberculosis* Genetic Diversity and Molecular Epidemiology. Infect. Genet. Evol. 12, 755–766. 10.1016/j.meegid.2012.02.004 22365971

[B10] European Centre for Disease Prevention and Control (2016). Molecular Typing for Surveillance of Multidrug-Resistant Tuberculosis in the EU/EEA January. Stockholm: ECDC.

[B11] Glynn J. R. Whiteley J. Bifani P. J. Kremer K. Van Soolingen D. (2002). Worldwide Occurrence of Beijing/W Strains ofMycobacterium Tuberculosis: A Systematic Review. Emerg. Infect. Dis. 8, 843–849. 10.3201/eid0805.020002 12141971 PMC2732522

[B12] Gurevich A. Saveliev V. Vyahhi N. Tesler G. (2013). QUAST: Quality Assessment Tool for Genome Assemblies. Bioinformatics 29, 1072–1075. 10.1093/bioinformatics/btt086 23422339 PMC3624806

[B13] Han S. J. Song T. Cho Y.-J. Kim J.-S. Choi S. Y. Bang H.-E. (2015). Complete Genome Sequence of *Mycobacterium tuberculosis* K from a Korean High School Outbreak, Belonging to the Beijing Family. Stand. Genomic Sci. 10, 78. 10.1186/s40793-015-0071-4 26473025 PMC4606834

[B14] Hillemann D. Kubica T. Agzamova R. Venera B. Rüsch-Gerdes S. Niemann S. (2005). Rifampicin and Isoniazid Resistance Mutations in *Mycobacterium tuberculosis* Strains Isolated from Patients in Kazakhstan. Int. J. Tuberc. Lung Dis. 9, 1161–1167. 16229229

[B15] Ibrayeva A. Kozhamkulov U. Raiymbek D. Alenova A. Igilikova S. Zholdybayeva E. (2014). Molecular Epidemiology of *Mycobacterium tuberculosis* Strains Circulating in the Penitentiary System of Kazakhstan [Short Communication]. Int. J. Tuberc. Lung Dis. 18, 298–301. 10.5588/ijtld.13.0558 24670565

[B16] Ilin A. I. Kulmanov M. E. Korotetskiy I. S. Akhmetova G. K. Lankina M. V. Shvidko S. V. (2015). Complete Genome Sequence of Multidrug-Resistant Clinical Isolate *Mycobacterium tuberculosis* 187.0, Used to Study the Effect of Drug Susceptibility Reversion by the New Medicinal Drug FS-1. Genome Announc 3. 10.1128/genomeA.01272-15 PMC4645197 26543112

[B17] Ioerger T. R. Koo S. No E.-G. Chen X. Larsen M. H. Jacobs W. R. Jr. (2009). Genome Analysis of Multi- and Extensively-Drug-Resistant Tuberculosis from KwaZulu-Natal, South Africa. PloS one 4, e7778. 10.1371/journal.pone.0007778 19890396 PMC2767505

[B18] Iwai H. Kato-Miyazawa M. Kirikae T. Miyoshi-Akiyama T. (2015). CASTB (The Comprehensive Analysis Server for the *Mycobacterium tuberculosis* Complex): A Publicly Accessible Web Server for Epidemiological Analyses, Drug-Resistance Prediction and Phylogenetic Comparison of Clinical Isolates. Tuberculosis 95, 843–844. 10.1016/j.tube.2015.09.002 26542225

[B19] Kaas R. S. Leekitcharoenphon P. Aarestrup F. M. Lund O. (2014). Solving the Problem of Comparing Whole Bacterial Genomes across Different Sequencing Platforms. PLoS One 9, e104984. 10.1371/journal.pone.0104984 25110940 PMC4128722

[B20] Kairov U. Kozhamkulov U. Molkenov A. Rakhimova S. Askapuli A. Zhabagin M. (2015). Draft Genome Sequences of Two Clinical Isolates of *Mycobacterium tuberculosis* from Sputum of Kazakh Patients. Genome Announc 3. 10.1128/genomeA.00466-15 PMC4432342 25977436

[B21] Kairov U. (2020). Whole-genome Sequencing and Bioinformatics Analysis of M.Tuberculosis Multi-Drug Resistant Strains from Kazakhstan. *ESHG 2020.2 - European Human Genetics Virtual Conference* [Online], P17 - Bioinformatics and Statistical Methods. Available: https://www.abstractsonline.com/pp8/#!/9102/presentation/1820 (Accessed June 6, 2020).

[B22] Kamerbeek J. Schouls L. Kolk A. Van Agterveld M. Van Soolingen D. Kuijper S. (1997). Simultaneous Detection and Strain Differentiation of *Mycobacterium tuberculosis* for Diagnosis and Epidemiology. J. Clin. Microbiol. 35, 907–914. 10.1128/jcm.35.4.907-914.1997 9157152 PMC229700

[B23] Köser C. U. Ellington M. J. Cartwright E. J. P. Gillespie S. H. Brown N. M. Farrington M. (2012). Routine Use of Microbial Whole Genome Sequencing in Diagnostic and Public Health Microbiology. PLoS Pathog. 8, e1002824. 10.1371/journal.ppat.1002824 22876174 PMC3410874

[B24] Kozhamkulov U. Akhmetova A. Rakhimova S. Belova E. Alenova A. Bismilda V. (2011). Molecular Characterization of Rifampicin- and Isoniazid-Resistant *Mycobacterium tuberculosis* Strains Isolated in Kazakhstan. Jpn. J. Infect. Dis. 64, 253–255. 21617314

[B25] Kubica T. Agzamova R. Wright A. Aziz M. A. Rakishev G. Bismilda V. (2005). The Beijing Genotype Is a Major Cause of Drug-Resistant Tuberculosis in Kazakhstan. Int. J. Tuberc. Lung Dis. 9, 646–653. 15971392

[B26] Kumar S. Stecher G. Li M. Knyaz C. Tamura K. (2018). MEGA X: Molecular Evolutionary Genetics Analysis across Computing Platforms. Mol. Biol. Evol. 35, 1547–1549. 10.1093/molbev/msy096 29722887 PMC5967553

[B27] Lanzas F. Karakousis P. C. Sacchettini J. C. Ioerger T. R. (2013). Multidrug-resistant Tuberculosis in panama Is Driven by Clonal Expansion of a Multidrug-Resistant *Mycobacterium tuberculosis* Strain Related to the KZN Extensively Drug-Resistant *M. tuberculosis* Strain from South Africa. J. Clin. Microbiol. 51, 3277–3285. 10.1128/jcm.01122-13 23884993 PMC3811646

[B28] Levaditi C. Vaisman A. Chaigneau H. (1950). Study of the Avirulent Strain H37Ra of *Mycobacterium tuberculosis* . C R. Seances Soc. Biol. Fil 144, 687–688. 14773133

[B29] Li H. Durbin R. (2009a). Fast and Accurate Short Read Alignment with Burrows-Wheeler Transform. Bioinformatics 25, 1754–1760. 10.1093/bioinformatics/btp324 19451168 PMC2705234

[B30] Li H. Handsaker B. Wysoker A. Fennell T. Ruan J. Homer N. (2009b). The Sequence Alignment/Map Format and SAMtools. Bioinformatics 25, 2078–2079. 10.1093/bioinformatics/btp352 19505943 PMC2723002

[B31] Mcarthur A. G. Waglechner N. Nizam F. Yan A. Azad M. A. Baylay A. J. (2013). The Comprehensive Antibiotic Resistance Database. Antimicrob. Agents Chemother. 57, 3348–3357. 10.1128/AAC.00419-13 23650175 PMC3697360

[B32] Mi H. Muruganujan A. Ebert D. Huang X. Thomas P. D. (2018). PANTHER Version 14: More Genomes, a New PANTHER GO-Slim and Improvements in Enrichment Analysis Tools. Nucleic Acids Res. 47, D419–D426. 10.1093/nar/gky1038 PMC6323939 30407594

[B33] Mitchell D. (2002). Book: Timebomb: The Global Epidemic of Multi-Drug Resistant Tuberculosis. BMJ : Br. Med. J. 324, 245a–245. 10.1136/bmj.324.7331.245a

[B34] Mokrousov I. Vyazovaya A. Solovieva N. Sunchalina T. Markelov Y. Chernyaeva E. (2015). Trends in Molecular Epidemiology of Drug-Resistant Tuberculosis in Republic of Karelia, Russian Federation. BMC Microbiol. 15, 279. 10.1186/s12866-015-0613-3 26679959 PMC4683759

[B35] Narayanan S. Deshpande U. (2013). Whole-Genome Sequences of Four Clinical Isolates of Mycobacterium tuberculosis from Tamil Nadu, South India. Genome Announc 1 **,** e00186-00113. 10.1128/genomeA.00186-13 23788533 PMC3707582

[B36] Phelan J. E. O’Sullivan D. M. Machado D. Ramos J. Oppong Y. E. A. Campino S. (2019). Integrating Informatics Tools and Portable Sequencing Technology for Rapid Detection of Resistance to Anti-tuberculous Drugs. Genome Med. 11, 41. 10.1186/s13073-019-0650-x 31234910 PMC6591855

[B37] Sekizuka T. Yamashita A. Murase Y. Iwamoto T. Mitarai S. Kato S. (2015). TGS-TB: Total Genotyping Solution for *Mycobacterium tuberculosis* Using Short-Read Whole-Genome Sequencing. PLoS One 10, e0142951. 10.1371/journal.pone.0142951 26565975 PMC4643978

[B38] Skiba Y. Mokrousov I. Ismagulova G. Maltseva E. Yurkevich N. Bismilda V. (2015). Molecular Snapshot of *Mycobacterium tuberculosis* Population in Kazakhstan: a Country-wide Study. Tuberculosis 95, 538–546. 10.1016/j.tube.2015.04.012 26076582

[B39] Soolingen D. v. De Haas P. E. W. Hermans P. W. M. Van Embden J. D. A. (1994). [15] DNA Fingerprinting of mycobacterium Tuberculosis. Methods Enzymol. 235, 196–205. 10.1016/0076-6879(94)35141-4 8057895

[B40] Supply P. Allix C. Lesjean S. Cardoso-Oelemann M. Rüsch-Gerdes S. Willery E. (2006). Proposal for Standardization of Optimized Mycobacterial Interspersed Repetitive Unit-Variable-Number Tandem Repeat Typing of *Mycobacterium tuberculosis* . J. Clin. Microbiol. 44, 4498–4510. 10.1128/jcm.01392-06 17005759 PMC1698431

[B41] Uddin R. Siddiqui Q. N. Azam S. S. Saima B. Wadood A. (2018). Identification and Characterization of Potential Druggable Targets Among Hypothetical Proteins of Extensively Drug Resistant *Mycobacterium tuberculosis* (XDR KZN 605) through Subtractive Genomics Approach. Eur. J. Pharm. Sci. 114, 13–23. 10.1016/j.ejps.2017.11.014 29174549

[B42] Valway S. E. Sanchez M. P. C. Shinnick T. F. Orme I. Agerton T. Hoy D. (1998). An Outbreak Involving Extensive Transmission of a Virulent Strain ofMycobacterium Tuberculosis. N. Engl. J. Med. 338, 633–639. 10.1056/nejm199803053381001 9486991

[B43] Wattam A. R. Abraham D. Dalay O. Disz T. L. Driscoll T. Gabbard J. L. (2014). PATRIC, the Bacterial Bioinformatics Database and Analysis Resource. Nucl. Acids Res. 42, D581–D591. 10.1093/nar/gkt1099 24225323 PMC3965095

[B44] World Health Organization (2020). Global Tuberculosis Report.

[B45] World Health Organization (2003). Guidelines for Surveillance of Drug Resistance in Tuberculosis. Third edition. Geneva.

[B46] World Health Organization (1998). Laboratory Services in Tuberculosis Control. Part III: Culture. Geneva.

[B47] World Health Organization (2017). Tuberculosis Epidemiological Impact Analysis and Assessment of TB Surveillance System of. Kazakhstan.

[B48] Yin Q.-Q. Liu H.-C. Jiao W.-W. Li Q.-J. Han R. Tian J.-L. (2016). Evolutionary History and Ongoing Transmission of Phylogenetic Sublineages of *Mycobacterium tuberculosis* Beijing Genotype in China. Sci. Rep. 6, 34353. 10.1038/srep34353 27681182 PMC5041183

[B49] Zankari E. Hasman H. Cosentino S. Vestergaard M. Rasmussen S. Lund O. (2012). Identification of Acquired Antimicrobial Resistance Genes. J. Antimicrob. Chemother. 67, 2640–2644. 10.1093/jac/dks261 22782487 PMC3468078

