# Supplementary material for: Genomic Analysis of Multidrug-Resistant Mycobacterium tuberculosis Strains From Patients in Kazakhstan
Source: Front Genet. 2021 Nov 9;12:683515. doi: 10.3389/fgene.2021.683515 (PMC8630622; doi:10.3389/fgene.2021.683515)
Supplement: Supplementary file 1 [file Table1.DOCX]

Supplementary Material

**Genomic analysis of multidrug resistant *Mycobacterium tuberculosis* strains from patients in Kazakhstan**

Asset Daniyarov, Askhat Molkenov, Saule Rakhimova, Ainur Akhmetova, Dauren Yerezhepov, Lyailya Chingissova, Venera Bismilda, Bekzat Toksanbayeva, Ainur Akilzhanova, Ulan Kozhamkulov* and Ulykbek Kairov^*^

*** Correspondence:** Ulykbek Kairov: ulykbek.kairov@nu.edu.kz; Ulan Kozhamkulov: ulan.kozhamkulov@nu.edu.kz

# Supplementary Data

**Supplementary file S1 – Variant calling pipeline.**

#!/bin/bash

# conda create -n kpipeline

# conda activate kpipeline

fastq**=$1**

prefix**=$(basename $fastq .fastq)**

ref**=**h37rv.fna

###########################################

# quality control

##########################################

# conda install -c bioconda fastqc=0.11.8

**mkdir** -p qc**/**${prefix}

**export** _JAVA_OPTIONS**=**"-Xmx4G"

fastqc ${fastq} -o qc**/**${prefix}

###########################################

# trimming adapters and low-quality bases

##########################################

# conda install -c bioconca trim-galore=0.6.4

**mkdir** -p fastq_trim**/**

trim_galore **-**q 20 -o fastq_trim**/** ${fastq}

###########################################

# quality control after trimming

###########################################

**mkdir** -p qc_trim**/**${prefix}

fastqc fastq_trim**/**${prefix}_trimmed.fq -o qc_trim**/**${prefix}

###########################################

# alignment

###########################################

# conda install -c bioconda bwa

**mkdir** -p aln**/**

bwa index ${ref}

bwa mem -M -t 20 ${ref} ${fastq} **>** aln**/**${prefix}**.**sam

###########################################

# sam to indexed bam

###########################################

# conda install -c bioconda samtools

samtools view **-**Sbh aln**/**${prefix}**.**sam **>** aln**/**${prefix}**.**bam

samtools sort aln**/**${prefix}**.**bam -o aln**/**${prefix}**.**sort.bam

samtools index aln**/**${prefix}**.**sort.bam

###########################################

# unique mappling reads [based on quality; -q 20]

###########################################

samtools view **-**bh **-**q 20 aln**/**${prefix}**.**sort.bam **>** aln**/**${prefix}**.**uniq.sort.bam

samtools index aln**/**${prefix}**.**uniq.sort.bam

###########################################

# duplicated reads removal

# -s (single-end)

###########################################

samtools rmdup -s aln**/**${prefix}**.**uniq.sort.bam aln**/**${prefix}**.**uniq.sort.rmdup.bam

samtools index aln**/**${prefix}**.**uniq.sort.rmdup.bam

###########################################

# bam to vcf

###########################################

# conda install -c bioconda bcftools

# apt install libssl-dev

samtools mpileup **-**gf ${ref} aln**/**${prefix}**.**uniq.sort.rmdup.bam **|** bcftools call **-**m **-**Ov -o ${prefix}**.**vcf

bcftools filter **-**i'%QUAL>20' ${prefix}**.**vcf **|** bcftools stats **|** **grep** TSTV

###########################################

# clean cumbersome data

###########################################

**rm** -f aln**/**${prefix}**.**bam aln**/**${prefix}**.**sam

# 6_102.sort.bam 6_102.uniq.sort.bam 6_102.uniq.sort.rmdup.bam

# 6_102.sort.bam.bai 6_102.uniq.sort.bam.bai 6_102.uniq.sort.rmdup.bam.bai

###########################################

# anounce completion

###########################################

**echo** "##############################"

**echo** "DONE!"

**echo** "##############################"
