# Supplementary material for: Genomic Analysis of Multidrug-Resistant Mycobacterium tuberculosis Strains From Patients in Kazakhstan
Source: Front Genet. 2021 Nov 9;12:683515. doi: 10.3389/fgene.2021.683515 (PMC8630622; doi:10.3389/fgene.2021.683515)

Supplementary Material

**Genomic analysis of multidrug resistant *Mycobacterium tuberculosis* strains from patients in Kazakhstan**

Asset Daniyarov, Askhat Molkenov, Saule Rakhimova, Ainur Akhmetova, Dauren Yerezhepov, Lyailya Chingissova, Venera Bismilda, Bekzat Toksanbayeva, Ainur Akilzhanova, Ulan Kozhamkulov* and Ulykbek Kairov^*^

*** Correspondence:** Ulykbek Kairov: ulykbek.kairov@nu.edu.kz; Ulan Kozhamkulov: ulan.kozhamkulov@nu.edu.kz

**Supplementary file S7 -** Functional analysis plot using GO with the PANTHER system. A - Fisher’s exact test; B - Binomial statistic.


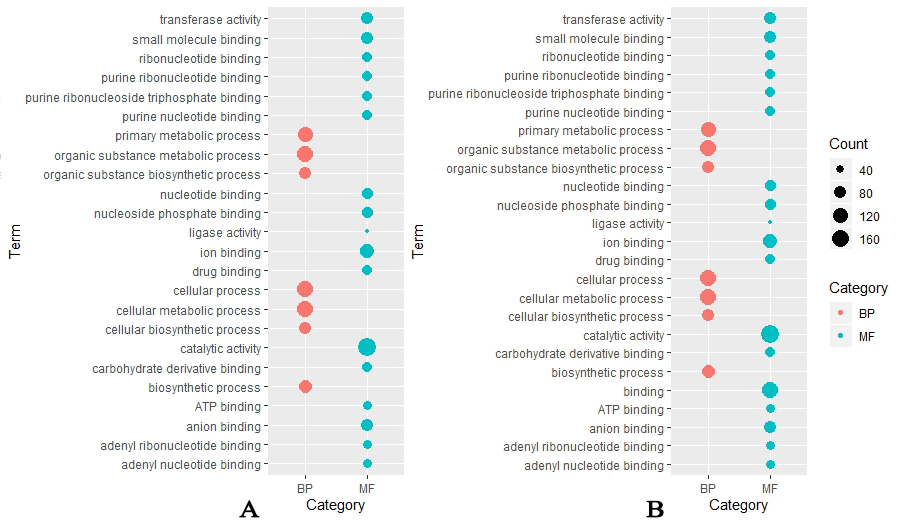

Supplement: Supplementary file 2 [file Table7.DOCX]
