# Supplementary material for: Genomic Analysis of Multidrug-Resistant Mycobacterium tuberculosis Strains From Patients in Kazakhstan
Source: Front Genet. 2021 Nov 9;12:683515. doi: 10.3389/fgene.2021.683515 (PMC8630622; doi:10.3389/fgene.2021.683515)
Supplement: Supplementary file 3 [file Table2.DOCX]

Supplementary Material

**Genomic analysis of multidrug resistant *Mycobacterium tuberculosis* strains from patients in Kazakhstan**

Asset Daniyarov, Askhat Molkenov, Saule Rakhimova, Ainur Akhmetova, Dauren Yerezhepov, Lyailya Chingissova, Venera Bismilda, Bekzat Toksanbayeva, Ainur Akilzhanova, Ulan Kozhamkulov* and Ulykbek Kairov^*^

*** Correspondence:** Ulykbek Kairov: ulykbek.kairov@nu.edu.kz; Ulan Kozhamkulov:

**Supplementary file S2 – R Script.**

#indels annotation

library(data.table)

library(readxl)

DT <- read_excel("GMTVD_Indels.xlsx")

library(vcfR)

DT1 <- read.table("20_1713.vcf", sep="\t")

setnames(COHORT, c("V1", "V2", "V3", "V4", "V5", "V6", "V7", "V8", "V9", "V10"), c("CHROM", "Position in genome", "ID", "REF", "ALT", "QUAL", "FILTER", "INFO", "FORMAT", "INFO_VCF"))

library(stringr)

library(stringi)

#true_var <- DT1[nchar(as.character(DT1$`2_1280.vcf`))>10, ]

DT3 <- merge(DT, DT1, by.x="Position in genome", by.y = "Position in genome") #all matches, but snps are also present

library(dplyr)

indels <- DT3 %>%

filter(str_detect(INFO, "^INDELS*"))

write.csv(indels, file = "20_1713_indels.csv") #only indels

min(nchar(as.character(indels$REF)))

max(nchar(as.character(indels$REF)))

min(nchar(as.character(indels$ALT)))

max(nchar(as.character(indels$ALT)))

#snps annotation

DT <- read_excel("GMTVD_snp.xlsx")

DT1 <- read.table("20_1713.vcf", sep="\t")

setnames(COHORT, c("V1", "V2", "V3", "V4", "V5", "V6", "V7", "V8", "V9", "V10"), c("CHROM", "Position in genome", "ID", "REF", "ALT", "QUAL", "FILTER", "INFO", "FORMAT", "INFO_VCF"))

DT3 <- merge(DT, DT1, by.x="Position in genome", by.y = "Position in genome")

snps <- DT3 %>%

filter(str_detect(INFO, "^DP*"))

write.csv(snps, file = "20_1713_snps.csv") #only snps

#stat

nns <- filter(snps, Effect == "Nonsyn") #all non-syn snps

s <- filter(snps, Effect == "Syn") #all syn snps

is <- filter(snps, Effect == "None") #all Intergenic snps

keep <- c("Gene")

DT4 <- nns[keep]

PPE <- nns[grep("^PPE", DT4[,1]), ] #all non-syn snps in PPE genes

PE <- nns[grep("^PE", DT4[,1]), ] #all non-syn snps in PE genes

DT5 <- snps[keep]

coding <- snps %>%

filter(str_detect(Gene, "^None")) # coding snps

coding <- snps[grep("None", DT5[,1]),] #or

l_snps <- c("Position in genome")

list_snps <- snps[l_snps]

write.csv(list_snps, file = "20_1713_snps_list.csv") #save snp as list, by each isolate

#########################

library(ggplot2)

library(readxl)

dataset = "Panther.xlsx"

#GO_Fisher's Exact

dataset = as.data.frame(read_excel(dataset, sheet = "GO_Fisher's Exact"))

head(dataset)

ggplot( dataset, aes(x=Category, y=Term, size = Count, colour = Category)) +

geom_point()

#GO_Binomial

dataset = as.data.frame(read_excel(dataset, sheet = "GO_Binomial"))

head(dataset)

ggplot( dataset, aes(x=Category, y=Term, size = Count, colour = Category)) +

geom_point()

##########################
