# Supplementary material for: Genomic Analysis of Multidrug-Resistant Mycobacterium tuberculosis Strains From Patients in Kazakhstan
Source: Front Genet. 2021 Nov 9;12:683515. doi: 10.3389/fgene.2021.683515 (PMC8630622; doi:10.3389/fgene.2021.683515)
Supplement: Supplementary file 4 [file Table3.DOCX]

Supplementary Material

**Genomic analysis of multidrug resistant *Mycobacterium tuberculosis* strains from patients in Kazakhstan**

Asset Daniyarov, Askhat Molkenov, Saule Rakhimova, Ainur Akhmetova, Dauren Yerezhepov, Lyailya Chingissova, Venera Bismilda, Bekzat Toksanbayeva, Ainur Akilzhanova, Ulan Kozhamkulov* and Ulykbek Kairov^*^

*** Correspondence:** Ulykbek Kairov: ulykbek.kairov@nu.edu.kz; Ulan Kozhamkulov: ulan.kozhamkulov@nu.edu.kz

**Supplementary file S3 - List of reference strains with countries and publications.**

RUS_B0 - Russian Federation (https://www.ncbi.nlm.nih.gov/pubmed/26679959)

XDR KZN 605 - South Africa (https://www.ncbi.nlm.nih.gov/pubmed/19890396)

PanR1006 - South Africa (https://www.ncbi.nlm.nih.gov/pubmed/23884993)

str. Beijing/NITR203 – India (https://www.ncbi.nlm.nih.gov/pubmed/23788533)

K - South Korea (https://www.ncbi.nlm.nih.gov/pmc/articles/PMC4606834/)

H37Rv – UK (https://www.ncbi.nlm.nih.gov/pubmed/9634230)

H37Ra – France (https://www.ncbi.nlm.nih.gov/pubmed/14773133)

CDC1551 – USA (https://www.ncbi.nlm.nih.gov/pubmed/12218036)

KZN 4207 – South Africa (https://www.ncbi.nlm.nih.gov/pubmed/19890396)
