# Supplementary material for: Genomic Analysis of Multidrug-Resistant Mycobacterium tuberculosis Strains From Patients in Kazakhstan
Source: Front Genet. 2021 Nov 9;12:683515. doi: 10.3389/fgene.2021.683515 (PMC8630622; doi:10.3389/fgene.2021.683515)
Supplement: Supplementary file 5 [file Table4.DOCX]

Supplementary Material

**Genomic analysis of multidrug resistant *Mycobacterium tuberculosis* strains from patients in Kazakhstan**

Asset Daniyarov, Askhat Molkenov, Saule Rakhimova, Ainur Akhmetova, Dauren Yerezhepov, Lyailya Chingissova, Venera Bismilda, Bekzat Toksanbayeva, Ainur Akilzhanova, Ulan Kozhamkulov* and Ulykbek Kairov^*^

*** Correspondence:** Ulykbek Kairov: ulykbek.kairov@nu.edu.kz; Ulan Kozhamkulov: ulan.kozhamkulov@nu.edu.kz

**Supplementary file S4 - Distribution of SNPs and Indels identified in antibiotic resistance-associated regions of *M.tuberculosis* between isolates (from ResFinder 3.2 and TB profiler)^a^**

| **Isolate** | **Known target: Amino Acid Mutation** | **Novel / unknown target: Amino Acid Mutation** |
| --- | --- | --- |
| MTB-MDR-KZ (1280) | katG: S315T (INH^1^), katG: R463L (FLQ^6^), rpoB: S450L (RIF^2^), embB: M306V (EMB^3^), gyrA: S95T (FLQ^6^),  rpsL: K43R (SM^5^) | thyA: P253A, gyrA: E21Q, G668D,  rpoC: E1092D, ethA: T314I, gidB: E92D, kasA: K34Q,  rpsA: R212R, tlyA: L11L, embC: R927R, embA: C76C |
| MTB-MDR-KZ (1405) | katG: S315T (INH^1^), katG: R463L (FLQ^6^), pncA: G24D (PZA^4^), rpsL: K43R (SM^5^), gyrB: D461N (FLQ^6^), rpoB: S450L (RIF^2^), embB: M306V (EMB^3^), gyrA: S95T (FLQ^6^) | embC: A594A, R927R, rpoB: H723D, rpoC: E1092D,  gidB: E92D, rrl r.1204delC, rpoB p.H723D,  gyrA: E21Q, G668D, ethA: T314I, thyA: P253A, gyrB p.K5K – Frameshift, gyrB p.K6R, gyrB p.K7R, gyrB p.A8P, gyrB p.Q9K, gyrB p.D10T, gyrB p.E11N, gyrB p.Y12T, gyrB p.G13A, gyrB p.A14L, gyrB p.A15R, gyrB p.S16L, gyrB p.I17S, gyrB p.T18P, gyrB p.I19F, gyrB p.L20S, gyrB p.E21K, gyrB p.L23W, gyrB p.E24R, gyrB p.A25P, gyrB p.V26S, gyrB p.R27A, gyrB p.K28N, gyrB p.R29V, gyrB p.G31A, gyrB p.M32C, gyrB p.Y33T, gyrB p.I34L, gyrB p.G35A, gyrB p.S36R, gyrB p.T37P, gyrB p.G38V, gyrB p.E39S, gyrB p.R40A, gyrB p.G41V, gyrB p.L42Y, gyrB p.H43T, gyrB p.H44I, gyrB p.L45S, gyrB p.I46F, gyrB p.W47G, gyrB p.E48R, gyrB p.V49W, gyrB p.V50S, gyrB p.D51T, gyrB p.N52T, gyrB p.A53R, gyrB p.V54S, gyrB p.D55T, gyrB p.E56R, gyrB p.A57R, gyrB p.M58W, gyrB p.A59P, gyrB p.G60V, gyrB p.Y61M, gyrB p.A62Q, gyrB p.T63P, gyrB p.T64Q, gyrB p.V65* - Premature stop codon |
| MTB-MDR-KZ (1410) | katG: S315T (INH^1^), katG: R463L (FLQ^6^), gyrB: D461N (FLQ^6^), rpoB: S450L (RIF^2^), embB: M306V (EMB^3^), rpsL: K43R (SM^5^), rpoC: N698S (RIF^2^), gyrA: S95T (FLQ^6^) | pncA: V155M, rpoC: E1092D, tlyA: L11L,  gyrA: E21Q, G668D, ethA: T314I, gidB: E92D, thyA: P253A, gyrB p.K5K – Frameshift, gyrB p.K6R, gyrB p.K7R, gyrB p.A8P, gyrB p.Q9K, gyrB p.D10T, gyrB p.E11N, gyrB p.Y12T, gyrB p.G13A, gyrB p.A14L, gyrB p.A15R, gyrB p.S16L, gyrB p.I17S, gyrB p.T18P, gyrB p.I19F, gyrB p.L20S, gyrB p.E21K, gyrB p.L23W, gyrB p.E24R, gyrB p.A25P, gyrB p.V26S, gyrB p.R27A, gyrB p.K28N, gyrB p.R29V, gyrB p.G31A, gyrB p.M32C, gyrB p.Y33T, gyrB p.I34L, gyrB p.G35A, gyrB p.S36R, gyrB p.T37P, gyrB p.G38V, gyrB p.E39S, gyrB p.R40A, gyrB p.G41V, gyrB p.L42Y, gyrB p.H43T, gyrB p.H44I, gyrB p.L45S, gyrB p.I46F, gyrB p.W47G, gyrB p.E48R, gyrB p.V49W, gyrB p.V50S, gyrB p.D51T, gyrB p.N52T, gyrB p.A53R, gyrB p.V54S, gyrB p.D55T, gyrB p.E56R, gyrB p.A57R, gyrB p.M58W, gyrB p.A59P, gyrB p.G60V, gyrB p.Y61M, gyrB p.A62Q, gyrB p.T63P, gyrB p.T64Q, gyrB p.V65* - Premature stop codon |
| MTB-MDR-KZ (1524) | katG: S315T (INH^1^), katG: R463L (FLQ^6^), rpoB: S450L (RIF^2^), embB: M306V (EMB^3^), pncA: L182S (PZA^4^), rpsL: K43R (SM^5^), gyrA: S95T (FLQ^6^) | rpoC: E1092D, V1039A, thyA: P253A, pncA: R176C, T177A, A178P, S179P, V180A, E181S, V183W, C184F, S185A, S186A, gyrA: E21Q, G668D, ethA: T314I, embR: p.C372G, embR: p.F376L, embR: p.Q379E, embR: p.I380V, pncA: p.M175_M175delinsMRT – Frameshift, gidB: p.E92D  embA: C76C |
| MTB-MDR-KZ (1525) | katG: S315T (INH^1^), katG: R463L (FLQ^6^), rpoB: S450L (RIF^2^), embB: M306V (EMB^3^), pncA: T76P (PZA^4^), rpsL: K43R (SM^5^), gyrA: S95T (FLQ^6^) | rpoC: E1092D, thyA: P253A, gyrA: E21Q, G668D, ethA: T314I, gidB p.E92D, rrl r.1846delC, embR p.C372G, embR p.F376L, embR p.Q379E, embR p.I380V, embA: C76C |
| MTB-MDR-KZ (1577) | katG: S315T (INH^1^), katG: R463L (FLQ^6^), rpoB: S450L (RIF^2^), embB: M306V (EMB^3^), rpsL: K43R (SM^5^), pncA: promoter n.-11 A>G (PZA^4^), gyrA: S95T (FLQ^6^) | katG: R463L, gyrA: E21Q, G668D, gidB: p.E92D,  thyA: P253A, rpoB: H723D, rpoC: E1092D, embC: R927R,  embA: C76C, |
| MTB-MDR-KZ  (1585) | katG: S315T (INH^1^), katG: R463L (FLQ^6^), rpoB: S450L (RIF^2^), embB: M306V (EMB^3^), rpsL: K43R (SM^5^), rrs: 1401 A>G (AMK^7^, CM^8^, KM^9^), gyrA: S95T (FLQ^6^) | rpoC: N698S, E1092D, thyA: P253A, gyrA: E21Q, G668D, pncA: T114R, V155M, rpsA: M432T, gidB: p.E92D |
| MTB-MDR-KZ (1713) | katG: S315T (INH^1^), katG: R463L (FLQ^6^), rpoB: S450L (RIF^2^), embB: M306V (EMB^3^), rpsL: K43R (SM^5^), pncA: T76P (PZA^4^), gyrA: S95T (FLQ^6^) | gyrA: E21Q, G668D, thyA: L11L, P253A, embC: R927R, fabG1: E76E – Frameshift, E77S, H78T, Q79R, G80V, P81R, V82S, E83R, V84C, L85W, V86C, S87P, N88T, A89P, G90A, L91Y, S92P, A93R, D94T, A95H, F96S, L97S, M98C, R99G, M100* - Premature stop codon, rpoC: E1092D, ethA: T314I, rrl: r.1079_1080insA, r.1130_1131insG, gidB: p.E92D, embR: p.E118E - Frameshift, p.T120N, p.A121R, p.V123R, p.H124A, p.A125R, p.A126G, p.A127R, p.A128R , p.G129R, p.R130P, p.F131V, p.E132R, p.Q133T, p.A134S, p.S135Q, p.R136P, p.H137P, p.L138P, p.S139V, p.A140G, p.A141R, p.L142I, p.R143E, p.E144R, p.W145M, p.R146A, p.G147W, p.P148A, p.V149G, p.L150A, p.D151R, p.D152* - Premature stop codon |

^a^ Abbreviations and symbol: ^1^ isoniazid - INH, ^2^ rifampicin - RIF, ^3^ ethambutol - EMB, ^4^ pyrazinamide - PZA, ^5^ streptomycin - SM, ^6^ fluoroquinolones - FLQ^6^;

Matches in two tools highlighted in color.
