# Supplementary material for: Genomic Analysis of Multidrug-Resistant Mycobacterium tuberculosis Strains From Patients in Kazakhstan
Source: Front Genet. 2021 Nov 9;12:683515. doi: 10.3389/fgene.2021.683515 (PMC8630622; doi:10.3389/fgene.2021.683515)
Supplement: Supplementary file 6 [file Table6.DOCX]

Supplementary Material

**Genomic analysis of multidrug resistant *Mycobacterium tuberculosis* strains from patients in Kazakhstan**

Asset Daniyarov, Askhat Molkenov, Saule Rakhimova, Ainur Akhmetova, Dauren Yerezhepov, Lyailya Chingissova, Venera Bismilda, Bekzat Toksanbayeva, Ainur Akilzhanova, Ulan Kozhamkulov* and Ulykbek Kairov^*^

*** Correspondence:** Ulykbek Kairov: ulykbek.kairov@nu.edu.kz; Ulan Kozhamkulov: ulan.kozhamkulov@nu.edu.kz

**Supplementary file S6 - Spoligotype, lineage and clade of the eight Kazakhstan *Mycobacterium tuberculosis* isolates.**

| Isolate | Drug susceptibility | Spoligotype^a^ | MIRU-VNTR profile (12 loci) | Lineage | Clade |
| --- | --- | --- | --- | --- | --- |
| MTB-MDR-KZ (1280) | INH - R; RIF - R  EMB - R; SM - S | 000000000003771 | 223325153533 | East Asian | Beijing |
| MTB-MDR-KZ (1405) | INH - R; RIF - R  EMB - R; SM - R | 000000000003771 | 223325173533 | East Asian | Beijing |
| MTB-MDR-KZ (1410) | INH – R; RIF - R  EMB - R; SM - R | 000000000003771 | 223325153533 | East Asian | Beijing |
| MTB-MDR-KZ (1524) | INH - R;RIF - R  EMB - R; SM - R | 000000000003771 | 223325153533 | East Asian | Beijing |
| MTB-MDR-KZ (1525) | INH - R; RIF - R  EMB - R; SM - R | 000000000003771 | 223325153533 | East Asian | Beijing |
| MTB-MDR-KZ (1577) | INH - R; RIF - R  EMB - R; SM - R | 000000000003771 | 223325153533 | East Asian | Beijing |
| MTB-MDR-KZ (1585) | INH - R; RIF - R  EMB - R; SM - R | 000000000003771 | 223325173533 | East Asian | Beijing |
| MTB-MDR-KZ (1713) | INH - R; RIF - R  EMB - R; SM - R | 000000000003771 | 223325153533 | East Asian | Beijing |

^a^ Expressed in octal format

INH - Isoniazid; RIF – Rifampicin; EMB - Ethambutol; SM - Streptomycin

R - resistant; S – susceptible
